# Stakeholder Perceptions of the Acceptability of Peer-Mediated Intervention for Minimally Speaking Preschoolers on the Autism Spectrum

**DOI:** 10.1007/s10803-023-05928-5

**Published:** 2023-03-01

**Authors:** Michelle O’Donoghue, Norelee Kennedy, John Forbes, Carol-Anne Murphy

**Affiliations:** 1 School of Allied Health, Faculty of Education and Health Sciences, Health Research Institute, University of Limerick, Limerick, V94 T9PX Ireland; 2 Graduate Entry Medical School, Faculty of Education and Health Sciences, Health Research Institute, University of Limerick, Limerick, Ireland

**Keywords:** Peer-mediated intervention, Autism, Minimally speaking, Acceptability, Stakeholders

## Abstract

Peer mediated intervention (PMI) is an evidence-based approach to supporting social and communication development for children on the autism spectrum. For PMI to be integrated into everyday practice, it needs to be acceptable to stakeholders. This article engaged with autistic individuals, early childhood educators, parents, and speech and language pathologists on the prospective acceptability of implementing PMI with minimally speaking preschoolers in inclusive preschool settings. Focus groups and semi-structured interviews were conducted. The transcriptions were analyzed qualitatively using reflexive thematic analysis. Stakeholders described PMI as an acceptable intervention approach for this population and provided valuable insights to inform the development and implementation of PMIs. Attention needs to be paid to how to support preschools to adopt a PMI-friendly philosophy.

There is a gap between autism research and its application in educational settings (Parsons & Kasari, 2013). Guldberg (2017) cautions against explaining this gap as a failure of practitioners to apply research and calls for the use of methodologies that position educators, autistic people, and their families at the center. Much of our knowledge about peer-mediated intervention (PMI) and minimally speaking children on the autism spectrum has been generated from efficacy studies, typically using a single case experimental design methodology. The stakeholder voice is largely absent. The current study engages with stakeholder groups to inform the development and implementation of PMI for minimally speaking children on the autism spectrum in real world preschool contexts.

In inclusive education settings, children with various diagnoses and language abilities play, explore, and learn together. One of the key tenets of inclusive education is that peers are a key source of learning. However, simply placing children on the autism spectrum into preschool settings, in the absence of specific supports and strategies, is unlikely to influence their play and interaction experiences (Gunn et al., 2014). This is particularly relevant for minimally speaking children, as peers may not know how to engage with a playmate who uses very little verbal language to communicate, or who uses an alternative and augmentative communication (AAC) device. Specific interventions are needed to promote positive social experiences for these children (Steinbrenner, 2018).

The goal of social communication interventions is for children to be able to interact in their natural social contexts (DiSalvo & Oswald, 2002), and so intervention frequently focuses on building the skills and capabilities of the child’s communication partners, most frequently their caregivers. Naturalistic interventions, with a parent education component, are the most well-researched approach with this population and appear to be effective at supporting expressive communication (Koegel et al., 2019) and joint engagement (Kasari et al., 2015) and research in early childhood contexts suggests that with sufficient training and coaching early childhood educators (ECEs) can support increased engagement in preschool for children on the autism spectrum (e.g., Boyd et al., 2018). Incorporating peers into interventions addressing social and communication skills fosters the creation of supportive communication partners and increases the likelihood that intervention gains will generalize beyond the treatment context (Hugh et al., 2021). This is a priority for parents, who want their children to build relationships with their peer group (O’Leary & Moloney, 2020).

PMI involves selecting peers close in age, and training and supporting them to interact with a child on the autism spectrum (Odom & Strain, 1984). Several reviews have concluded that PMI promotes social interaction between children on the autism spectrum and their peers (Chan et al., 2009; Ledford & Pustejovsky, 2021), including reviews focusing on group studies (Chang & Locke, 2016), preschool age children (Chapin et al., 2018), inclusive preschool settings (Watkins et al., 2015), and children with autism who are minimally speaking (O’Donoghue et al., 2021). O’Donoghue et al. (2021) identified twelve single case experimental design studies that used PMI and were conducted with minimally speaking preschoolers on the autism spectrum (*n* = 28) and their typically developing peers (*n* = 60). In these studies, interventionists were typically researchers and/or ECEs. The interventionists sought to teach the peers and/or minimally speaking participants on the autism spectrum interaction strategies, for example, to establish mutual attention, talk about ongoing activities and respond to their communication partner (e.g., Goldstein et al., 1992), and/or the peers learned how to use the child’s communication device (e.g., Thiemann-Bourque et al., 2017). The interventionists supported participants to use these strategies in play sessions varying in length from five to twenty minutes, two to three times per week. Following the introduction of PMI, children were found to spend more time interacting with one another and take more communicative turns in these interactions (O’Donoghue et al., 2021).

PMI for minimally speaking children on the autism spectrum can be incorporated into the natural preschool context and routine (Trembath et al., 2009) and has the potential to increase social interaction in inclusive preschool settings without requiring intensive training, nor extensive adult involvement (Watkins et al., 2019). It can achieve generalization to non-treatment settings and communication partners (Watkins et al., 2015). Non-autistic peer participants report that they enjoy the experience, have a better understanding of their autistic peers, and have developed friendships while taking part in PMI (Chan et al., 2009; Odom, 2019; Simpson & Bui, 2016). Despite this, PMI is utilized by little over half of special educators on a regular basis (Knight et al., 2019) and preschool teachers feel that it is not age appropriate (Hugh et al., 2021).

For PMI to be integrated into everyday practice, it needs to be acceptable to stakeholders, including autistic people, ECEs, parents, and related professionals. Acceptability is the extent to which people who are delivering or receiving an intervention consider it to be appropriate, based on anticipated or experienced responses to the intervention (Sekhon et al., 2017). It includes the feelings that stakeholders have about the intervention, the perceived burden or effort they associate with it, how it aligns with their value system and how confident they feel to implement it (Sekhon et al., 2017). There has been limited exploration of prospective acceptability in relation to PMI. Social validity data gathered post-intervention indicate that preschool staff view PMI positively, but that it is challenging to implement in everyday practice with minimally speaking children on the autism spectrum (Lee & Lee, 2015). It is not clear what modifications to PMI are needed to best support children with complex communication needs (Ledford & Pustejovsky, 2021). Developing interventions in collaboration with stakeholders and end-users increases the usability of interventions, thus maximizing their effectiveness (Dingfelder & Mandell, 2011). It allows for the examination of contextual factors and practical considerations, thus facilitating alignment across research and implementation contexts (Carrington et al., 2016; Glasgow et al., 2019; McCreight et al., 2019). The aim of the present study is to engage with stakeholders to explore the prospective acceptability of implementing PMI for minimally speaking preschoolers on the autism spectrum.

## Methods

### Study Design

This is a qualitative study using focus groups and semi-structured interviews following the Consolidated Criteria for Reporting Qualitative Studies (COREQ) reporting guidelines (Tong et al., 2007; see Appendix A for checklist) and Ethical Code for Early Childhood Researchers (EECERA; Bertram et al., 2016). We aimed to collect the perspectives of stakeholders across various roles (ECEs, Speech and Language Pathologists (SLPs)) and contexts (autistic adults, parents of minimally speaking children and parents of non-autistic preschoolers) to better understand the acceptability of PMI from a variety of viewpoints (Aarons & Palinkas, 2007; Carey & Asbury, 2012). Focus groups allow for interactive discussion, with the potential for group interaction to prompt greater engagement with a topic (Carey & Asbury, 2012). Focus groups can be challenging for autistic people, as attending a new place, and interacting with new people can be anxiety-inducing (Gowen et al., 2019). Therefore, individual semi-structured interviews were used to gather the views of autistic adults. The interviews took place in a location of the participants’ choosing. Accommodations as recommended by Pellicano et al. (2017) were made, including providing the interview guide in advance, giving plenty of warning of changes to the setting or schedule and allowing the interviewee time to process what had been said before repeating a question. The data were analyzed qualitatively using reflexive thematic analysis (Braun & Clarke, 2006, 2019). This is the first study to focus solely on stakeholder views on peer-mediated intervention with minimally speaking children, and reflexive thematic analysis facilitated the researchers to adopt an inductive approach and work within a constructivist epistemology (consistent with Braun & Clarke, 2022). This study design was reviewed and accepted by the University of Limerick, Faculty of Education and Health Sciences Research Ethics Committee (2019_06_26_EHS). *.*

Each focus group comprised of only one category of stakeholder, i.e., all ECEs, all parents of minimally speaking preschoolers on the autism spectrum, all parents of non-autistic preschoolers and all SLPs. This was to facilitate the disclosure of attitudinal or organizational influences on behavior that may be challenging to disclose to a mixed group (Carey & Asbury, 2012).

The context for the current study was the Republic of Ireland where there is a universal two-year preschool program (Department of Children, Equality, Disability, Integration and Youth, 2021). The program is provided for three hours per day, five days per week over 38 weeks per year. A model of support is currently in place to ensure that children with disabilities can access the preschool program. SLPs are predominantly employed in health settings and provide input to preschool children directly and indirectly in clinical and educational contexts, working with parents, educators and others as required.

Focus groups with ECEs and SLPs progressed as planned, as did face-to-face interviews with autistic participants. COVID-19 restrictions were introduced before the focus groups with parents had been conducted. Early childhood education settings and schools were closed. Some workplaces were closed, and others remained open. Face to face meetings were prohibited. This was the first lock down in the Republic of Ireland and it was a time of uncertainty for many people. To facilitate the continuation of the study, we altered our data collection approach for these groups to the use of online, recorded, one-on-one semi-structured interviews. This was preferable to online focus groups as it was challenging for people to commit to a specific day and time in advance, so individual interviews allowed for more flexibility as interviews could be scheduled at a time that suited each participant. These changes were reviewed and approved by the ethics committee.

### Sample

We sought to recruit participants from across five stakeholder groups: autistic adults, ECEs, parents of autistic children who are or were minimally speaking during the preschool years (i.e., using significantly fewer words than expected levels relative to age, as per Koegel et al., 2020), parents of non-autistic children who currently attend preschool and SLPs. The inclusion criteria required that participants were living in the Republic of Ireland, could express themselves using the English language and that ECEs and SLPs had at least 12 months experience working with preschoolers on the autism spectrum, to ensure the inclusion of sufficiently experienced participants. There is no sample size requirement for reflexive thematic analysis research (Braun & Clarke, 2022), instead sample size is determined by a mix of interpretative, situated, and pragmatic judgements (Braun & Clarke, 2019). We planned to conduct one focus group per stakeholder group. We estimated a provisional sample size of between 20 and 60 participants, based on focus group guidelines of 4–12 participants per focus group (Tong et al., 2007). Participants for semi-structured interviews and focus groups were invited via purposive and convenience sampling through the following channels: the national SLP Autism Special Interest Group (SIG), local preschool network, local parent organizations, and the university’s autism SIG. Emails were distributed via contact points in each organization.

Nineteen participants, across five stakeholder groups participated: autistic adults (*n* = 3), ECEs (*n* = 5), parents of autistic children who are or were minimally speaking during the preschool years (*n* = 4), parents of non-autistic children who currently attend preschool (*n* = 2), and SLPs (*n* = 5). Characteristics of the participants are provided in Table 1.

**Table 1:** Participant characteristics

Stakeholder group			
Autistic adults	Age range	History of being minimally speaking (MS)	Method of data collection
Autistic adult 1	20–29	Yes	Individual semi-structured interviews conducted in person
Autistic adult 2	30–39	No	
Autistic adult 3	30–39	No	

Early childhood educators (ECEs)	Age range	Years of experience as ECE	Method of data collection
ECE 1	50–59	28	Focus Group conducted in person in local hotel meeting room
ECE 2	40–49	30	
ECE 3	50–59	20	
ECE 4	40–49	23	
ECE 5	40–49	13	

Speech and language pathologists (SLPs)	Age range	Years of experience as SLP	Method of data collection
SLT 1	20–29	4.5	Focus Group conducted in person in local work organization’s meeting room
SLT 2	20–29	5	
SLT 3	30–39	8	
SLT 4	20–29	1.5	
SLT 5	50–59	30	

Parents of MS autistic children	Age range	Age of child/ren and current verbal status	Method of data collection
Parent of MS child 1	40–49	6 years (not MS)	Individual semi-structured interviews conducted online
Parent of MS child 2	40–49	11 years, 9 years and 5 years (not MS)	
Parent of MS child 3	30–39	3 years (MS)	
Parent of MS child 4	40–49	7 years (MS)	

Parents of non-autistic (NA) preschoolers	Age range	Age of child	Method of data collection
Parent of NA child 1	30–39	3 years	Individual semi-structured interviews conducted online
Parent of NA child 2	40–49	4 years	

Before COVID-19 restrictions were introduced, five parents had signed up to each of the two planned parent’s focus groups. When contacted again post-COVID-19 restrictions, one parent of a minimally speaking autistic child did not respond, leaving a total of four participants in this stakeholder group. Of the five parents of typically developing preschoolers, three indicated by return email that they no longer had availability to participate in the study, leaving a total of two participants in this stakeholder group. We deemed it relevant to include the groups with smaller representation in the analysis as this is an exploratory study (Carey & Asbury, 2012), consideration of this topic is novel, and it was important to include these stakeholder perspectives. Our sample size of 19 is just short of our provisional sample size range, and balancing the impact of COVID-19 and the pragmatics of recruitment we are confident the data add to an understanding of this topic area.

The interviewer (first author) was known to six of the participants prior to study commencement due to her previous involvement with the groups in the various recruitment channels. All participants were aware that the interviewer was conducting this study in part fulfilment of her PhD, as per the Information Sheet (see Appendix B). Participants received no financial remuneration. Tea/coffee/water and light refreshments were available for participants where focus groups/interviews were face-to-face.

Several participants (*n* = 5) met the inclusion criteria for more than one stakeholder group, highlighting the multiplicity of people’s experiences, and the challenges in separating people into different stakeholder groups. Participants were only counted as members in the group to which they had contributed, that is, no participants were double counted. One of the ECEs and one of the autistic adults are parents to minimally speaking children on the autism spectrum. An autistic adult participant holds a qualification in early childhood education, as does one of the parents of a minimally speaking child. Finally, one of the parents of a minimally speaking child also has a non-autistic preschooler. If the researcher knew this information in advance, the participant was offered a choice as to which stakeholder group they would like to contribute to.

### Researcher Reflexivity

The researchers engaged in reflexive practice throughout the research process, interrogating and considering their positionality and influence (as per Holmes, 2020). The first author, an SLP with a Master of Science degree, has worked in preschool settings as an SLP for four years, and as a lecturer in SLP for 6 years. She is an active member of local and national autism SIGs. Her understanding of autism is shaped by the children and families with whom she has worked, her autistic collaborators, friends, and their families, and the concept of neurodiversity. She views inclusive education opportunities in a positive light and has research experience in qualitative methodologies including focus groups and interviews.

The final author, an SLP with a Doctoral Degree and wide research experience, has worked as a SLP with children with communication difficulties and as a lecturer and researcher in speech and language therapy for 19 years. She has particular interests in intervention development and implementation for children with communication difficulties including valid approaches for educational settings.

Both second and third authors are senior academics with extensive research experience across multiple methodologies. Both are active in intervention development and implementation.

### Procedure

The Participant Information Sheet (Appendix B) was supplied to potential participants during recruitment. Participants also had an opportunity to read the information sheet at the beginning of the focus group/interview and to ask questions. If the participants were happy to proceed, then they were provided with the consent form. No participants dropped out or decided not to participate at this stage.

The first author conducted the interviews and focus groups. The final author monitored the audio-recordings for adherence to the study procedure and engaged in transcript checking and theme development.

Focus groups lasted for 40 min (SLP) and 60 min (ECE) and were audio-recorded. Participants were encouraged to respect the privacy of others by agreeing not to disclose anything about the focus group discussion to others. Semi-structured interviews lasted from 10 to 40 min (median = 29 min) and were audio-recorded. One interview was particularly short (10 min). This interview was with an autistic participant, who had considered the interview script in advance and provided answers to all the questions asked. Their rate of speech is fast and there was little deviation from the questions in the interview script. When engaging with people from various sociocultural backgrounds it can be necessary to challenge some of the beliefs that we hold about what is appropriate or sufficient when using qualitative methods (Chauhan & Sehgal, 2022). The first author conducted each interview. The face-to-face focus groups and interviews took place in a space mutually agreed between the participant(s) and researcher and consisted of a mix of workplace and neutral environments (see Table 1). Only the participants and researchers were present. The online interviews were conducted using Microsoft Teams.

Focus Groups and Interviews followed guides (see Appendix C). These guides were informed by Duda's implementation science in education framework (Duda & Wilson, 2018) and the contextual factors identified in the Practical, Robust Implementation and Sustainability Model (PRISM) by McCreight et al. (2019). The guides were also influenced by the systematic review of PMI with minimally speaking children on the autism spectrum conducted by the current research team (O’Donoghue et al., 2021)*.* There were slight differences in the questions that were put to the professionals (SLPs and ECEs) and to parents and autistic adults. This was to allow for the exploration of issues that are of particular relevance to each group, for example, the lived experience of an autistic adult. There were no further differences in the guides across focus groups and interviews. All guides began with the first author providing a description of PMI and asking participants whether they had experience of this intervention. No participants had been involved in a PMI as described.

The first author transcribed the focus groups/interviews verbatim. Participants were assigned pseudonyms to protect anonymity. Once the audio recording of each focus group/interview was transcribed, verified (four transcripts were double checked for procedural and transcription accuracy by the final author), and cleaned (participant identifiers removed), the original audio recordings were permanently deleted. Short, keyword field notes were handwritten during the interviews and focus groups and stored with study data.

### Analytical Approach

Braun and Clarke’s (2021) six-phase analytical process was used: (1) data familiarization; (2) systematic data coding; (3) generating initial themes; (4) developing and reviewing themes; (5) refining, defining, and naming themes; and (6) writing the report. In the initial stages of the analysis, the first author engaged in an in-depth reading of the transcripts to become attuned to key issues and ideas arising from the perspectives of participants. NVivo software was used to manage the dataset. A semantic approach to coding was adopted in order to describe the experiences of the participants, with themes considered in terms of their broader meaning and implications (Braun & Clarke, 2006). In reflexive thematic analysis coding quality is not dependent on multiple coders, and a single analyst is common (Braun & Clarke, 2022). Forty-six data codes were identified during systematic data coding. These were organized into preliminary thematic areas. The first and last author discussed and developed these themes over a series of three meetings. The meetings facilitated the primary analyst (first author) to enhance interpretative depth and for authors to ensure that alternative interpretations and possibilities were considered. Conversations centered around reflecting on the inductive nature of the analysis, ensuring that theme generation was not the provision of a summary of the answers provided to the questions asked during the focus groups/interviews and that codes that challenge predominant views in the literature on PMI were addressed. Candidate themes were identified that captured how some codes were clustering together. This is a subjective and interpretative process in which the authors take an active role in knowledge production (Braun & Clarke, 2019). These themes were reviewed and analyzed until each theme was clearly defined. Analytical memos were created within the software to document key insights and decisions made, as a strategy to enhance the overall trustworthiness and transparency of the analytical process. The first author prepared a Microsoft Word document detailing each theme, with illustrative examples from the text. This was shared with co-authors and discussed in a peer debriefing session. Transcripts were not returned to participants to comment on, but a summary of the themes was shared with participants.

## Results

All stakeholder groups expressed an openness to utilizing peer-mediated approaches in preschool settings to support the learning and inclusion of minimally speaking children on the autism spectrum. Building the peers’ skills was perceived as a means to reduce pressure on the child on the autism spectrum to conform and to facilitate an “evenness” (ECE 1) in interaction, while also helping to cultivate a sense of belonging in the preschool setting. Stakeholders identified challenges to introducing an intervention approach that represents a “paradigm shift” (parent of non-autistic (NA) child 1), moving from a sole focus on the child on the autism spectrum, to thinking about the role of other children in the environment.

**Fig. 1:**
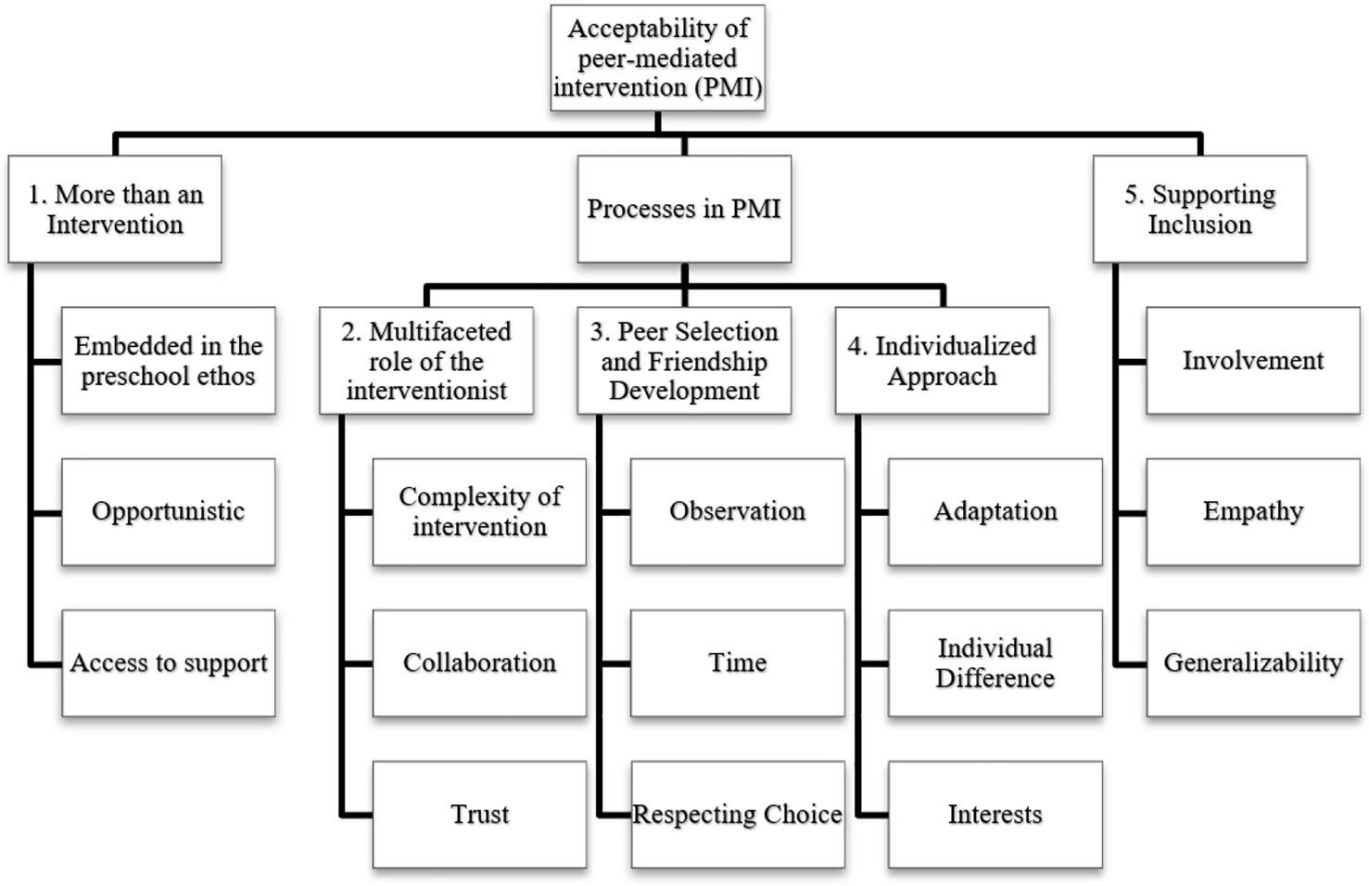
Coding tree, top

Five themes were generated through analysis of the data (summarized in Fig. 1). The first theme ‘more than an intervention’ represents an organizational and attitudinal approach to PMI. The second, third and fourth themes relate to the implementation of PMI: the multifaceted role of the interventionist, peer selection and its relation to natural friendship development, and an individualized approach. The fifth theme ‘supporting inclusion’ relates to observable changes because of involvement in PMI. All stakeholder groups have at least one statement assigned to each identified theme.

### More Than an Intervention

PMI was conceptualized as being embedded within the preschool’s philosophy, rather than as a stand-alone intervention that takes place at specific times of the day. It needs to become the “norm” for the preschool and “part of their ethos” (parent of NA child 1). It is not a “nice, charitable thing to do”, it is “fulfilling the (autistic) child’s human rights” (autistic adult 3).

If your peers and those around you were taught how to interact with you and how to support you and how to meet you where you are (you) wouldn’t have to … go the full way, you could meet at halfway. All of the work wouldn’t be on one side. (Autistic adult 3).

To achieve this, staff need to be “on board”, if not “they’ll do it every so often … and then it just won’t work” (autistic adult 2).

Staff need to engage in both “opportunism and orchestration” (parent of minimally speaking (MS) child 1), setting up PMI at pre-determined points during the preschool day, but also taking advantage of naturally occurring opportunities as they arise, “tweaking what's there naturally” (parent of NA child 1). Participants described how an observer would not notice PMI overtly: “It wouldn’t stand out, it would just be like the other interactions taking place in the room: the playing, the disagreements, the turn taking, the crying, the laughing … you wouldn’t really notice that this is something that’s going on” (autistic adult 3). This removes any need to single out the child on the autism spectrum, “that’s the worst thing, to single them out” (autistic adult 1).

Embedding PMI in this fashion could negate the need to wait for specialist support to implement it. Participants described “missing the key opportunity” (SLP 1) to intervene early and waiting a significant amount of time for a diagnosis “there’s children coming in … and there’s a year there of a gap before they’re getting assessed” (ECE 2). Nevertheless, outside of specialist services, a lack of understanding of autism limits the ability of preschool staff and parents to successfully implement strategies such as PMI: “In the last five years the children that have come into our service that are non-speaking has increased … and we’re only learning now really to change our ways” (ECE 3). Without specialist support there is an “insecurity” (parent of MS child 3) in decision-making and a “fear of doing the wrong thing” (autistic adult 3).

### Multifaceted Role of the PMI Interventionist(s)

A complex picture emerged of the role of the adult(s) responsible for implementing PMI, typically identified as a preschool staff member. The level of parental support or buy-in will depend on the trust that parents have in the person telling them about PMI, “if you have a parent in a good place, they’re much more open then when you’re coming with a suggestion, saying ‘I’d like to try this’ or ‘I’d like to try that’, they’re less cautious of you” (ECE 5). Trust must also exist between the PMI interventionist and the children involved, “that relationship has to be there between the teacher and the child” (parent of MS child 4); “trust has to be built up” (ECE 3).

When implementing PMI, the adults responsible need to set up opportunities for interaction, while also facilitating naturally occurring interactions as they arise. They provide support for both the child on the autism spectrum and the peer(s) in the interaction, modify this support depending on the situation, modify the intervention to suit an individual child’s needs, explain the children’s behavior, provide praise, and also coach the children through when the interaction “fails” (parent of MS child 1). This occurs in the context of collaboration and consultation with other team members, including the child themselves, caregivers, and specialists. The PMI interventionist needs to communicate how PMI is progressing to other team members and understand that change may be “a slow process” (ECE 2) and not “an overnight thing” (autistic adult 3). Beyond direct PMI itself, preschool staff also have an active role in supporting their colleagues to engage in PMI and providing encouragement in order “to keep that morale up” (ECE 5).

Participants warned against PMI being the remit of one member of staff, or a specialist member of staff (e.g., Special Needs Assistant), while also proposing appointing a PMI “champion” (autistic adult 2) who would build their own knowledge and expertise of PMI and then support other staff members in its implementation. Staff training is necessary to build capacity and understanding: “if it was to be done properly, you have to have the teacher trained” (parent of MS child 4), “there’s a lot of training involved obviously of staff” (SLP 2), “you need a level of expertise for a child … to do justice for the child, to do justice to the parent” (ECE 5). Currently, training opportunities are limited “[there is] so little support for preschool educators both in terms of training and the cost” (autistic adult 3).

### Peer Selection and Friendship Development

Stakeholders described peer selection for PMI as happening over time, mirroring natural friendship development. Early childhood educators described letting children “find their own way” (ECE 4), and how all children may be drawn to particular peers, “certain children gravitated straight away … the children who naturally gravitated were the children who wanted to be there … and they gravitated not because they saw the child as different, they gravitated because they like something about that child” (ECE 5). Similarly, the child on the autism spectrum may also choose the children with whom they want to interact.

We had one little boy, he would eat his lunch at the windowsill, and…over time … he graduated bit by bit and would stand at the edge of the table before he sat down. But, he still picked the table he liked the children at. You know, so then we said, ok now we have a group, now we have a group to work with. (ECE 4)

At times, this process may involve adult involvement and trial and error, “You would try and see people that you think would match together, personality wise, and try and get them in the same area. Maybe with a similar activity that they enjoyed. And try to see would it happen organically” (ECE 1).

The parents of minimally speaking children cautioned against relying solely on one special friend “the one person that he seems to click with” (parent of MS child 4), as over time “friendships may drift apart” (parent of MS child 1), and the autistic child may become isolated. Parents proposed that extending the number of children who understand their child’s behavior would lead to greater participation in preschool activities. The parent of a non-autistic preschooler hypothesized that peers would influence one another and seeing one child engaging in PMI would lead other children to seek it out.

### Individualized Approach

Participants foregrounded the importance of individualizing and adapting PMI to meet the needs of each child. “Every one of them is going to be different” (parent of MS child 4) so “[PMI] has to be tailored to the child’s abilities and what the child wants” (autistic adult 2). Children may be ready for different levels of peer interaction, and the kind of support that they need in these interactions may vary, for example, “it may be a very sociable child, who perhaps needs … some boundaries put in place” (autistic adult 3). Children’s receptive language level and sensory requirements will influence the implementation of PMI. One parent described how their child finds noisy environments challenging, and therefore PMI with their child would need to be facilitated in a quiet environment, separate to the main preschool space (parent of MS child 2).

Children also need to have a choice to opt-out, “maybe preschool is already overwhelming enough” (autistic adult 3) and “we don’t know what the kid thinks” (parent of a MS child 1). This may involve some “trial and error” (autistic adult 3), as there may be particular times of day that suit the child better than others do and “there will be days when you just can’t do it” (autistic adult 2). Engaging in PMI may be tiring for the autistic child:

I think you've … got to understand just how much of a challenge it is for the child that is speaking or non-speaking, that it potentially may take a whole lot out of them. It may be that after that they feel like they want to be on their own for 45 minutes, and that’s okay. (Parent of MS child 2)

One avenue to individualize PMI is to incorporate the child on the autism spectrum’s interests. This can provide an avenue for communication (“the reason I didn’t communicate is because I wasn’t interested in what they were talking about”), connection (“I would have liked to be involved more if the people were interested in what I was interested in … I would have liked to connect with them”) and shared enjoyment (“they love talking about their things”) (autistic adult 1). This could involve individualizing the activities used, for example, setting up a pretend play opportunity based on pirates for one child, but for another child it is based on zoo animals. This engagement with the child’s interest was described as a “nice bridge” (parent of MS child 2), while the parents of non-autistic preschoolers predicted that once the activity was play-based it would be of sufficient interest to their child to hold their attention.

The PMI interventionist would benefit from open communication with other stakeholders to identify how best to individualize the approach and to discuss challenges that arise: “We took advice from everyone, and we kind of tweaked it a little bit ourselves as well, and it worked” (parent of MS child 3).

### Supporting Inclusion

Participants identified potential positive changes (both immediate and long term) for both the minimally speaking children and their non-autistic peers, because of their involvement in PMI.

For the child on the autism spectrum, participants described them as experiencing an increased level of inclusion “bringing them into the social circle” (parent of NA child 2), and their enjoyment of this inclusion “[being] there with others and happy to be there” (parent of MV child 4). This has potential long-term benefits: “he’s not going to be alone in his life. The more he is exposed to others the better” (parent of MS child 1). Increased interaction presents an opportunity for learning language from their peers, and for learning generally, “it would bring them on” (autistic adult 2). This learning is mutual “she learns from them, and they learn from her” (parent of MS child 3).

For the peers, stakeholders identified PMI as an opportunity to develop empathy, “put themselves in the other child’s shoes” (SLP 2) and to learn about people’s differences, “the more knowledge that they have about differences then they’re … more comfortable” (SLP 1). PMI also provides chances to advance children’s social skills, “teaching a child social skills to interact with a child that is < on the autism spectrum > … is going to aid the child learning social skills in the rest of their social interactions as well” (parent of NA child 1). These skills have the potential to generalize to other settings, such as the playground, “the hope is that they’ll bring that with them going forward” (SLP 1). Parents would need information about how to make this happen, “if they were to be at a playground with their child, and they bumped into a child with additional needs…these skills are generalized … as parents can you support your child to deal with that situation?” (Parent of NA child 1).

Benefits may be experienced at a societal level as peers become more understanding and accepting of difference, “one of the main [barriers] is the judgement and attitudes of others … there is a huge benefit to other people being more open minded” (autistic adult 3). PMI provides a way to address this societal barrier “this is a nice way to plant the seed in parents of young children … that … it’s their children [who] are going to be the ones to support anybody with extra needs in the society” (parent of NA child 1). The youthful status of the children was important in this regard “it’s a lot harder to teach them the older they get, whereas if they are brought up with it … it’s going to be a lot easier” (autistic adult 2). One parent of a non-autistic preschooler described their child’s acceptance of the “different” behavior of their friend who is on the autism spectrum.

He was at a party here a few months back, it was very noisy, there were kids everywhere going bananas and he couldn’t cope, and he had to go home. And the lads are just like, ‘that’s Billy, he doesn’t like noise, you know’. So, he’s a little different but they don’t have any bias or anything towards him, you know. That’s the good thing about that age group … they don't recognize differences as much as older groups you know? (Parent of NA child 2)

One parent identified capturing these changes as a “challenge [because PMI] is about the relationship” and so it may need consideration “over time” (parent of NA child 1).

## Discussion

These stakeholders consider PMI to be an acceptable intervention approach for minimally speaking children on the autism spectrum and their peers, and a potential positive addition to their preschool experience. Participants conceptualized PMI as an approach to be embedded in a preschool’s ethos, thereby reflecting that PMI is a good fit with stakeholder’s existing values. The role of the PMI interventionist was assigned to an ECE, and while a complex picture of this role was generated, ECE participants appeared confident in their ability to lead PMI with adequate training provision. Linkages were made between PMI and the existing preschool practices of supporting friendship development and individualizing support. Participants perceived PMI as potentially effective in supporting inclusion, with positive outcomes anticipated for the minimally speaking children and their peers.

The conceptualization of PMI as embedded in a preschool’s ethos and daily practice is more complex than the PMI often represented in the literature (for an exception see Goldstein et al., 1997). Typically, PMI with minimally speaking preschoolers on the autism spectrum is an intervention carried out at predetermined times of the day by a trained interventionist, who is frequently a member of the research team (O’Donoghue et al., 2021). In the current study, stakeholders perceived PMI as a failure if it was implemented in this manner. Instead, its value lies in how it can become part of the daily routine, “It’s not rocket science. This is just something that you have to learn to do and [it] will become part of everyday practice” (autistic adult 3). Its strength is that preschool staff can implement it throughout the day, as opportunities arise, and achieving this implementation goal will likely enhance the sustained impact of PMI (Odom, 2019). This reflects Guldberg’s (2017) observation that educators need to do more than simply apply a strategy, they need to understand it, know how to implement it in their particular context and reflect and adapt as necessary. Further stakeholder engagement could elucidate how to embed PMI in preschools and the impact this might have on PMI implementation (e.g., fidelity, dosage, duration, frequency, and effectiveness). Evaluation will require a sensitive observation tool that captures the communication environment and the ECE’s knowledge, skills, and approach, such as a modified version of the Communication Supporting Classrooms Observation Tool (Dockrell et al., 2015) which includes three dimensions: the language learning environment, language learning opportunities and language learning interactions. In addition to recording peer interactions and how ECEs support these interactions, such a tool could identify how the environment is set up to facilitate peers to interact with one another and could include documentation outlining the preschool’s approach to inclusion, thus capturing the ethos of the preschool.

The role of the PMI interventionist is complex. Stakeholders describe the strong relationships that this person must have with the parents and children involved, and the skills that they need to implement a specialist intervention in a way that is flexible and individualized. The complexity of this role is rarely considered in the intervention literature, perhaps partly because the researcher is frequently the interventionist (Thiemann-Bourque et al., 2016, 2017; Trembath et al., 2009), and partly because of the limited inclusion to date of stakeholders in the PMI design process. In studies where preschool educators lead PMI with minimally verbal autistic preschoolers there is no outline of how much training and support the educators require (Kohler et al., 1995, 2007; Odom & Watts, 1991). ECEs and SLPs report feeling poorly trained to meet the needs of young autistic children (Mullis, 2021; Rivard et al., 2015). The training needs of staff to implement PMI need to be made explicit. This will facilitate stakeholders to advocate for the system-level change needed to support PMI, for example, access to funding for specialist training. While formal training (e.g., professional development, seminars) may provide useful information, many ECEs and SLPs prefer learning “on the floor” in real time in the preschool, with support from their colleagues (Trembath et al., 2019).

Stakeholder engagement facilitates in-depth consideration of complex processes that may not be addressed comprehensively in intervention research, for example, peer selection in PMI studies. The most common method for choosing which peers will participate in PMI for minimally speaking preschoolers is ECE recommendation (O’Donoghue et al., 2021). A checklist of child characteristics may be outlined, for example, regular school attendance, age-appropriate social communication skills, and no negative social history with the child on the autism spectrum (Lee & Lee, 2015, based on criteria outlined by Strain & Odom, 1986). Interest surveys may be used to match children (Barber et al., 2016), and/or data from direct observation, caregiver reports and language assessments may be used (Trembath et al., 2009). However, it remains uncertain why one peer is chosen before another. Discussions around peer selection in the current study elucidate a complex process that aligns with natural friendship development. ECEs spend time observing which children naturally gravitate towards one another and aim to foster these connections and support continued interaction. This process takes time. PMI may be used to develop these burgeoning connections. Selecting peers with whom a child has a natural affinity may prove fruitful for potential generalization beyond the intervention context, foregrounding the potential role of PMI in friendship development (e.g., English et al., 1997; Vidal et al., 2018). Schwab (2017) reported that freely chosen contact with a peer with special educational needs in a secondary school setting was associated with positive attitudes towards peers with a disability. As recommended by DiSalvo and Oswald (2002), more explicit reporting is needed to understand what influences ECEs in their peer selection decisions, and to determine whether different approaches to peer selection affect PMI study outcomes.

In the current study, participants described PMI as tailored to meet the unique needs of individual children. Decisions on participation in the approach, and on the selection of activities, materials, settings, and peer partners can be informed by child preferences and strengths. Activities can be used that are engaging and reinforcing for all participants. Adapting PMI to suit the children on the autism spectrum’s interests creates a platform from which to authentically share an interaction and is in line with reported increases in social behavior when interests are incorporated into social activities (Baker, 2000; Boyd et al., 2007; Watkins et al., 2019). This establishes commonality, and a basis for communication and learning from each other. A struggle exists between the need to standardize intervention protocols for research purposes (ensuring that the intervention is consistently delivered and contains the necessary components) versus the need for flexibility in intervention delivery to meet individual needs. Single case experimental designs (SCEDs) dominate the intervention literature in PMI, and to some extent, this research design allows for individualization (e.g., see the description of individualized interest-based play activities used by Watkins et al., 2019). Nonetheless, what remains missing in the existing research base is a description of how researchers individualize PMI based on the participants that they encounter (both autistic and non-autistic) (Huber & Carter, 2016). Clear reporting is needed, including case studies detailing individualization in PMI, alongside experimental evaluation of individualized and non-individualized PMI to facilitate educators, clinicians, and parents to understand how they can use this approach to meet their child’s individual needs.

PMI research has primarily focused on quantitative measures of behavior change of the MV child on the autism spectrum and their peer, for example, the number of communicative turns, or the length of time that the child remains in an interaction (O’Donoghue et al., 2021). While important, these skills do not necessarily indicate that peer relationships have improved (Watkins et al., 2017). There has been insufficient focus on the experiences of the children (Ezzamel & Bond, 2016). According to the stakeholders in the current study, PMI could potentially increase inclusion, belonging and wellbeing. Embedding PMI within a preschool setting may reduce the need to single out children on the autism spectrum, a key strength of this approach for one autistic participant, echoing the findings of Bradley (2016) who reported that autistic students did not enjoy being singled out for interventions in primary school. Researchers need to report on whether the minimally speaking child on the autism spectrum is more settled and happier in their surroundings because of PMI. Is PMI worth the potential cognitive strain that it may place on the children taking part (both autistic and non-autistic)? Stakeholders identified PMI outcomes as increased empathy in peers and the potential for generalization of skills to new settings and different children. While these outcomes are not easy to measure, particularly with a minimally speaking population, they are important and worth more detailed consideration, as are the potential drawbacks for non-autistic participants such as missing out on engaging in alternative activities (Brock et al., 2018) and feeling unsure how to respond to the behavior of the child on the autism spectrum (Jones, 2007). Prioritizing stakeholder involvement in the early stages of intervention design will support the development of PMI research that measures the outcomes that matter most to parents and autistic people, including happiness, well-being, and quality of life (McConachie et al., 2015).

### Limitations and Future Directions

In an effort to appreciate the acceptability of PMI from a variety of viewpoints, participants from a range of stakeholder groups (autistic adults, ECEs, parents, SLPs) were invited to participate. The authors make no claims as to the sample being representative, but there are certain characteristics of the participants that may affect the interpretation of the results. None of the participants of the current study has participated in PMI, and their perceptions of its acceptability may change if they were to experience it. That said, prospective, or anticipated, acceptability remains worthy of investigation as it highlights aspects of an intervention that can be modified to enhance acceptability, and thus participation (Sekhon et al., 2021). The focus of the current study is on minimally speaking children on the autism spectrum, but only one of the three autistic participants had experience of being minimally speaking. The ECEs were all highly experienced. Perhaps less experienced ECEs would not have the confidence to take on the complex role of PMI interventionist, and this may act as a barrier to participation in PMI. The first author was known to six of the participants. While this is not inherently problematic (Carey & Asbury, 2012) it is possible that this prior relationship may have influenced the participants’ responses. The views of preschool children, both autistic and non-autistic, are not represented here. Children have important and highly relevant ideas and insight about their life experiences that we can learn from (Lyons et al., 2022). The authors intended to conduct a small group workshop with four preschool children using vignettes to explore the children’s ideas about interacting with children who have complex communication needs. Due to the onset of COVID-19 the workshop was not conducted, and the research team agreed that moving the session to an online forum was inappropriate given the young age of the participants, and the multimodal nature of the planned workshop.

According to the participants in the current study, PMI is an approach that is embedded in a preschool’s philosophy, and led by members of the preschool team, who implement it as opportunities arise throughout the school day. They described PMI as facilitating inclusion, and the children as learning from one another. This conceptualization may be better described as peer-mediated *accommodations*, rather than peer-mediated *intervention*, which may assume that the non-autistic children’s social interaction skills are in some way superior to the children on the autism spectrum. In line with guidance on developing complex interventions (O'Cathain et al., 2019), we propose conducting intervention development workshops with stakeholders to generate a protocol for an embedded PMI that is cognizant of implementation challenges and supports. Further investigation is required to determine whether this approach to PMI is as efficacious as more traditional approaches. This will involve data-gathering at multiple levels, including consideration of the preschool’s ethos and the communication environment, individual staff skills and knowledge, approaches to peer involvement and measuring the benefits as well as the costs to the children involved. Continued work with stakeholders may not only inform PMI development and implementation but may also reshape and reconfigure what is prioritized in PMI, in light of stakeholder views.

## Conclusions

PMI for minimally speaking children on the autism spectrum is an acceptable intervention to autistic adults, parents, ECEs and SLPs. Qualitative methodologies, such as those employed in the current study, position stakeholders at the center, support the examination of complexities that exist in designing interventions, and inform the implementation of PMI research in real-world contexts. Attention needs to be paid to how to support preschools to adopt a PMI-friendly philosophy, and investigation is needed as to whether this approach is as effective as more traditional PMI. More detailed reporting is needed on staff training requirements, peer selection processes, individualization strategies, and outcomes that reflect the desires of stakeholders. Our findings illustrate how stakeholder engagement provides valuable insights into PMI.

## Appendix A

**COREQ (Consolidated criteria for REporting Qualitative research) Checklist** developed from Tong A, Sainsbury, P., & Craig, J. (2007). Consolidated criteria for reporting qualitative research (COREQ): A 32-item checklist for interviews and focus groups. *International Journal for Quality in Health Care*, *19*(6), 349–357. https://doi.org/10.1093/intqhc/mzm042

**Table:**

Topic	Item no	Guide questions/description	Location
*Domain 1: Research team and reflexivity*			
*Personal characteristics*			
Interviewer/facilitator	1	Which author/s conducted the interview or focus groups	Method
Credentials	2	What were the researcher’s credentials? E.g., PhD, MD	Method
Occupation	3	What was their occupation at the time of the study?	Method
Gender	4	Was the researcher male or female?	Method
Experience and training	5	What experience or training did the researcher have?	Method
*Relationship with participants*			
Relationship established	6	Was relationship established prior to study commencement?	Method
Participant knowledge of the interviewer	7	What did the participants know about the researcher? E.g., personal goals, reasons for doing the research	Method
Interview characteristics	8	What characteristics were reported about the interviewer/facilitator? E.g., bias, assumptions, reasons and interests in the research topic	Method
*Domain 2: Study design*			
*Theoretical framework*			
Methodological orientation and Theory	9	What methodological orientation was stated to underpin the study? E.g., grounded theory, discourse analysis, ethnography, phenomenology, content analysis	Method
*Participant selection*			
Sampling	10	How were participants selected? E.g., purposive, convenience, consecutive, snowball	Method
Method of approach	11	How were participants approached? E.g., face-to-face, telephone, mail, email	Method
Sample size	12	How many participants were in the study?	Method
Non-participation	13	How many people refused to participate or dropped out? Reasons?	Method
*Setting*			
Setting of data collection	14	Where was the data collected? E.g., Home, clinic, workplace	Method
Presence of non-participants	15	Was anyone else present besides the participants and researchers?	Method
Description of sample	16	What are the important characteristics of the sample? E.g., demographic data	Method
*Data collection*			
Interview guide	17	Were questions, prompts, guides provided by the authors? Was it pilot tested?	Method and Appendix C
Repeat interviews	18	Were repeat interviews carried out? If yes, how many?	N/A
Audio/visual recording	19	Did the researcher use audio or video recording to collect the data?	Method
Field notes	20	Were field notes made during and/or after the interview or focus group?	Method
Duration	21	What was the duration of the interviews or focus group?	Method
Data saturation	22	Was data saturation discussed?	N/A The concept of data saturation is incompatible with a reflexive thematic analysis approach as adopted in this paper (see Braun & Clarke, 2019)
Transcripts returned	23	Were transcripts returned to participants to comment and/or correction?	Method
*Domain 3: Analysis and findings*			
*Data analysis*			
Number of data coders	24	How many data coders coded the data?	Method
Description of the coding tree	25	Did authors provide a description of the coding tree?	Results
Derivation of themes	26	Were themes identified in advance or derived from the data?	Method
Software	27	What software, if applicable, was used to manage the data?	Method
Participant checking	28	Did participants provide feedback on the findings?	Method
*Reporting*			
Quotations presented	29	Were participant quotations presented to illustrate the themes/findings? Was each quotation identified? E.g., participant number	Results
Data and findings consistent	30	Was there consistency between the data presented and the findings?	Results
Clarity of major themes	31	Were major themes clearly presented in the findings?	Results
Clarity of minor themes	32	Is there a description of diverse cases or discussion of minor themes	Results No distinction made specifically of minor themes, but examples of diverse views embedded within the consideration of each theme, reflecting real-world complexity

## Appendix B: Information Sheet

### Stakeholder Views on Peer-Mediated Intervention

#### Introduction

You are invited to take part in a study to explore using peer-mediated approaches in preschools. Peer-mediated approaches involve teaching non-autistic children strategies to support them to communicate with their autistic peers. This can help to increase the interaction between autistic children and their peers and create more opportunities for language learning and social interaction. We are particularly interested in children who use few or no words to communicate (i.e., are minimally speaking).

Currently, this approach is not widely used, and we would like to explore why this is the case, and to get some ideas to inform the development of a peer-mediated intervention for preschools.

We want to gather opinions and ideas from different viewpoints in order to create a resource that best reflects the values of those involved, and that is also appropriate for the context.

Through this study the research team aims to:

- Identify barriers to implementing peer-mediated intervention with minimally speaking autistic children in preschools and their peers
- Identify supports for such an approach
- Identify the desired outcomes of a peer-mediated intervention from a variety of stakeholders
- Inform the development of a resource and guidelines that could be used to support peer-mediated intervention in preschool environments.

#### Who Is on the Research Team?

*[Authors]*

#### Who Can Participate?

We are seeking people to take part in a number of Focus Groups or interviews.

Interviews: Adults (aged over 18) who have a diagnosis of autism.

Focus Group 1: Speech and Language Pathologists (SLPs) who have at least one year's experience working with minimally speaking autistic preschoolers.

Focus Group 2: Preschool Educators who have at least one year's experience of working in a preschool setting.

Focus Group 3: Parents whose children are/were minimally speaking during their preschool years (i.e. at ages 3, 4 and/or 5).

Focus Group 4: Parents whose children are non-autistic and are currently attending preschool.

#### What Does It Involve?

*Focus Groups*

- Focus groups will last up to 75 min and will be audio recorded.
- Focus groups will be held in a private space either on the *[university]* campus or a mutually preferred space.
- The number of people in a focus group will vary from three to eight.
- The researcher will take notes during the focus group and summarize the notes at the end of the session to ensure that all your key points were understood and documented.
- After the focus group the recording will be written out word for word and all identifying information will be removed. Participants will be given made up names to protect their privacy.

##### Interviews

- Interviews will last up to 45 min and will be audio recorded.
- Interviews will be held in a private space either on the *[university]* campus or a mutually preferred space.
- The researcher will take notes during the interview and summarize the notes at the end of the session to ensure that all your key points were understood and documented.
- After the interview the recording will be written out word for word and all identifying information will be removed. Interviewees will be given made up names to protect their privacy.

#### Benefits and Risks

There may be no direct benefits to you from taking part in this study; however, the information you provide will help to inform the development of peer-mediated approaches. There are minimal risks associated with taking part in this study. However, you may feel some discomfort if you are remembering some challenging times that you yourself, or your child had in preschool. If you foresee any accommodations that could assist you in taking part in this study, such as needing more time to answer questions, or having a preferred location, then please let the research team know as they would like to accommodate you to the best of their ability.

#### How Will Data Be Protected?

The Data Joint Controllers are the *[university]* and *[author]*. All information in relation to the study will be stored in a locked cabinet in the *[university]* for 7 years. At that point all hard copies and electronic data will be completely destroyed. All audio recordings and transcriptions will be downloaded onto a password protected computer. The University will not disclose your personal data to third parties. Further information on Data Protection at the *[university]* may be viewed at *[university webpage]*. You can contact the Data Protection Officer at *[university email]* or by writing to *[university]*.

#### How Are the Results Shared?

The focus group and interview information will be analyzed and written up by *[author]* in part fulfilment of the requirements of her doctoral studies at the *[university]*, under the supervision of *[authors]*. She will also make a narrated PowerPoint slide highlighting key points from the project to share via social media. Findings may also be presented at an international conference and published in a peer reviewed journal.

#### How to Find Out More and Participate

If you are interested in learning more about the study, or in taking part please contact *[author]*.

If you have concerns about this study, please contact the *[university]*.

## Appendix C: Focus Group and Interview Guides

All focus groups and semi-structured interviews will begin with an introduction to peer-mediated intervention and an operational definition of minimally speaking.

“I would like to outline some definitions of the terms that we are using in this focus group/interview. Pre-schoolers refer to children who are not yet attending primary school and are aged between 3 and 6. Minimally speaking is a term used to describe someone who uses few or no words to communicate. They could use another form of communication to help them to be understood such as gestures, a communication board or book or a communication app. And for the purposes of this study peer-mediated intervention is an approach where peers who share a pre-school space with an autistic child learn skills to help them to communicate with them. I will give you a few examples to show you what I mean:

1. Anna is 3 years old and has recently started at preschool. She is autistic. Her peers receive training with some tips for how to communicate with her. For example, they learn that they should try to stay in an area with Anna and share some space with her. They also learn that they can play alongside her, sometimes imitating what she is doing, and that they can talk to her by telling her what is happening. Adults set up play opportunities so that the peers have opportunities to use these skills and they prompt the children to use their strategies and praise them when they do.
2. Tommy is 4 years old and is autistic. He recently started to use a communication app on an iPad to provide an additional means for him to communicate with others. As well as Tommy learning how to use his device, his preschool peers learn how to use it too. Adults set up communication opportunities so that Tommy and his peers can communicate using his device. The peers are prompted and praised by the adults to use the device.

Approaches like these may be positive because they result in more social interaction for autistic preschoolers. They are also associated with more use of an AAC device. By creating more opportunities where the child is exposed to language and social interaction it may also help to create a language rich learning environment.

However, these approaches are not used all that much and my questions today will tease that out a little but more.”

## Focus Group Guides for Speech and Language Pathologists and Educators

### Opening Question (Round Robin Style for Everyone to Answer)

Do you currently use peer-mediated approaches?

If yes—can you share some more about that?

#### Key Questions

Thinking about the minimally speaking autistic children that you work with—could this kind of approach add any value to the work that you already do?

What would be the potential outcomes of using a peer-mediated approach?

Do you see this as part of your role?

Is there anyone else who you think should be involved in doing peer-mediated intervention?

Can you think of any factors or elements that would make this way of working difficult? (Barriers).

Can you think of any factors or elements that would support this way of working?

Do you feel confident in using a peer-mediated approach?

Why? Why not?

What would make you more confident?

What factors would you need to keep in mind if you were developing a peer-mediated intervention approach?

If a peer-mediated intervention was working really well—what would that look like?

#### Ending Question (Round Robin Style for Everyone Can Answer):

Any other comments?

### Semi-Structured Interview Guide for Parents

#### Opening Question

Are you aware of your child being involved in an approach like this before?

If so, can you tell me some more about it?

#### Key Questions

Thinking about your child/children, would this kind of approach have any added value?

What would the potential outcomes of a peer-mediated approach be?

Who do you think should be involved in this kind of approach?

Can you think of any factors or elements that would make this way of working difficult? (Barriers).

Can you think of any factors or elements that would support this way of working?

What factors would you need to keep in mind if you were developing a peer-mediated intervention approach?

If a peer-mediated intervention was working really well—what would that look like?

#### Ending Question

Any other comments?

### Semi-Structured Interview Guide for Autistic Adults

Were you involved in any approaches such as this?

Thinking about your early childhood years, would an approach such as this have had any added value?

What would the potential outcomes of a peer-mediated approach be?

Who do you think should be involved in this kind of approach?

Can you think of any factors or elements that would make this way of working difficult? (Barriers).

Can you think of any factors or elements that would support this way of working?

What factors would you need to keep in mind if you were developing a peer-mediated intervention approach?

If a peer-mediated intervention was working really well—what would that look like?

Any other comments?
